# Hsp78 (78 kDa Heat Shock Protein), a Representative AAA Family Member Found in the Mitochondrial Matrix of *Saccharomyces cerevisiae*

**DOI:** 10.3389/fmolb.2017.00060

**Published:** 2017-08-23

**Authors:** Josielle Abrahão, David Z. Mokry, Carlos H. I. Ramos

**Affiliations:** Chemistry Institute, University of Campinas Campinas, Brazil

**Keywords:** ATPases associated with diverse cellular activities, disaggregase, heat shock protein, molecular chaperones, protein folding and misfolding, heat shock protein 78 (HSP78), ClpB

## Abstract

ATPases associated with diverse cellular activities (AAA+) form a superfamily of proteins involved in a variety of functions and are characterized by the presence of an ATPase module containing two conserved motifs known as Walker A and Walker B. ClpB and Hsp104, chaperones that have disaggregase activities, are members of a subset of this superfamily, known as the AAA family, and are characterized by the presence of a second highly conserved motif, known as the second region of homology (SRH). Hsp104 and its homolog Hsp78 (78 kDa heat shock protein) are representatives of the Clp family in yeast. The structure and function of Hsp78 is reviewed and the possible existence of other homologs in metazoans is discussed.

## Introduction

**Figure 1:**
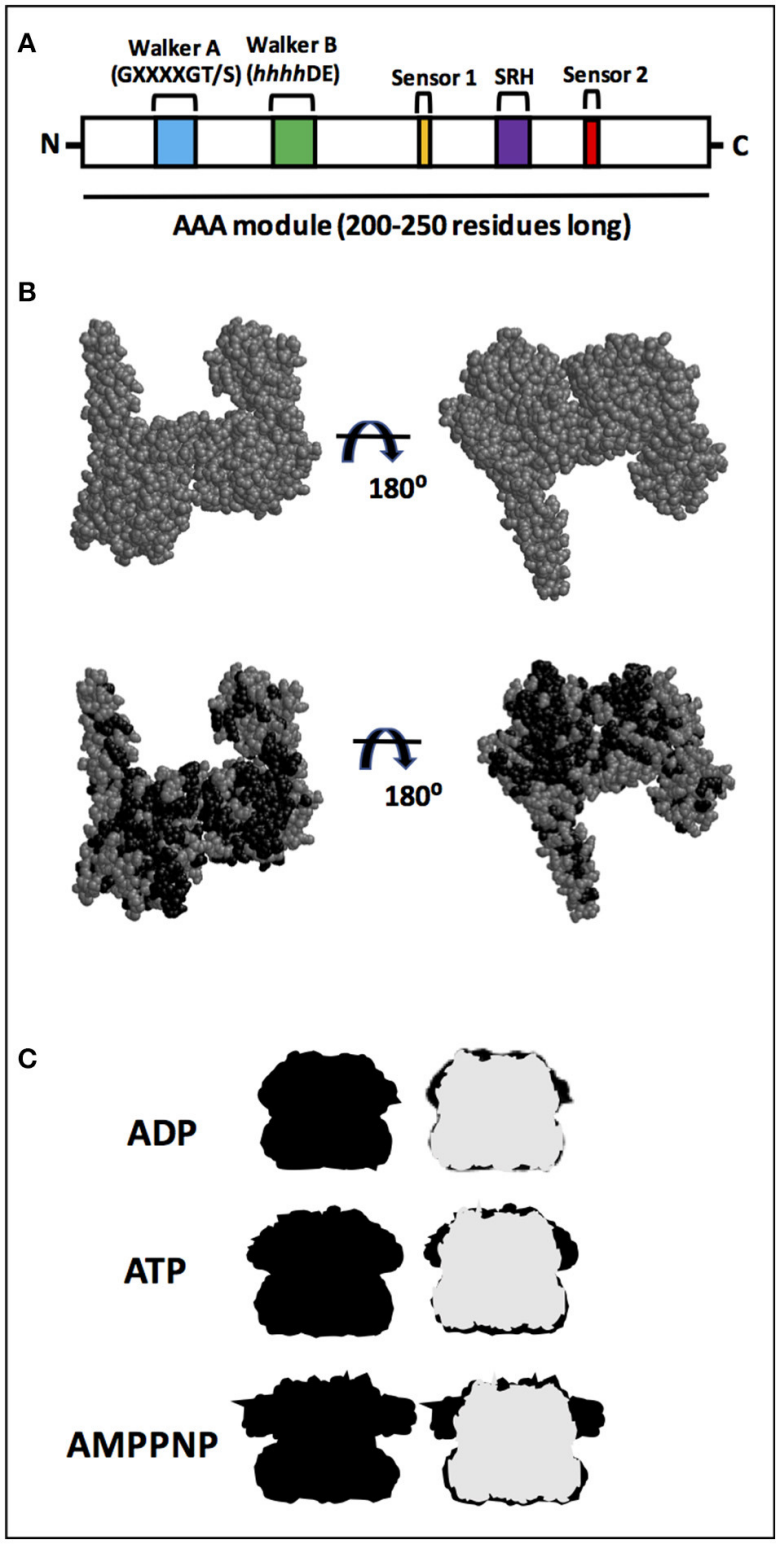
AAA+ superfamily features. **(A)** Proteins belonging to the AAA+ superfamily are characterized by the presence of an ATPase module which is 200–250 residues long with two motifs known as Walker A and Walker B. Additional motifs present are sensor 1 and sensor 2. A highly conserved motif, the second region of homology (SRH), defines the AAA family, a subset of the AAA+ family. **(B)** Structure from EcClpB (PDB number 4CIU; Carroni et al., 2014) from X-ray diffraction, which has a 3.5 Å resolution. Model for the 4CIU structure, in which all residues that are identical between EcClpB and Hsp78, according with the alignment shown in Figure S1, are colored in black. **(C)** Silhouette shape of hexameric ClpB from Cryo-EM. Shapes were drawn using Cryo-EM structures from Wendler and Saibil (2010). ClpB forms a ring-shaped hexameric structure in which the NBD1s from each monomer are on the top and the NBD2s are on the bottom. The structures are in the absence (APO; gray) and in the presence of ADP, ATP, or non-hydrolyzable AMPPNP (all in black). Superimposition with the apo form (right) indicates that the main conformational change upon either ATP or non-hydrolyzable AMPPNP binding occurs in the NBD1.

ATPases associated with diverse cellular activities (AAA+) form a superfamily of proteins involved in a variety of functions, from DNA replication to protein degradation (for reviews see Patel and Latterich, 1998; Sauer et al., 2004; Snider and Houry, 2008; Zolkiewski et al., 2012). Proteins belonging to the AAA+ superfamily are characterized by the presence of an ATPase module, which is 200–250 residues long containing two highly conserved motifs known as Walker A and Walker B and both interact with the bound nucleotide (Figure 1A). The Walker A motif (also known as the P-loop) is primarily responsible for binding ATP and has the consensus sequence GXXXXGK(T/S) (Walker et al., 1982), in which X is any residue and terminates with either a threonine or a serine residue. The Walker B motif is involved in hydrolyzing the bound nucleotide, and has the consensus sequence *hhhh*DE, in which *h* is a hydrophobic residue (Hanson and Whiteheart, 2005). Additional motifs present are sensor 1, a polar residue (usually asparagine) and sensor 2 (usually an arginine residue), and both are important for ATPase activity (Takahashi et al., 2002; Hanson and Whiteheart, 2005).

Moreover, a subset of the AAA+ family, known as the AAA family, possess a highly conserved motif called the second region of homology (SRH), which is ~15 residues long and has an arginine (arginine finger) involved in interunit interaction (Figure 1A) (Lupas and Martin, 2002). The AAA family is very large, including several clp members that are involved in remodeling target proteins (Hanson and Whiteheart, 2005). Among the members of the clp proteins within the AAA family are ClpB and Hsp104, well known chaperones which have disaggregase activities that can solubilize aggregates (for reviews see Shorter, 2008; Zolkiewski et al., 2012; Mokry et al., 2015). These aggregates are soluble or insoluble non-physiologically associations of misfolded proteins via exposed hydrophobic regions that are strongly correlated with diseases (for reviews see Ramos and Ferreira, 2005; Chiti and Dobson, 2006; Doyle et al., 2013; Knowles et al., 2014).

Additionally, even though members of the ClpB/Hsp104 subfamily are not essential under non-stress conditions, they confer protective qualities against diverse forms of stress. ClpB from bacteria *Escherichia coli* (EcClpB) and Hsp104 from yeast *Saccharomyces cerevisiae* (ScHsp104) are about 43% identical (Sanchez and Lindquist, 1990; Squires et al., 1991; Krzewska et al., 2001a; Figure S1). Despite these proteins both having two nucleotide binding domains (NBDs), called NBD1 and NBD2, they have limited homology between them (Schirmer et al., 1996). Indeed, one of the most well characterized Hsp100s, ScHsp104 was identified more than 20 years ago, as a stress-induced chaperone vital for tolerance to heat and ethanol stresses, and some heavy metals (Sanchez and Lindquist, 1990; Parsell et al., 1991; Lindquist and Kim, 1996). This protein is localized in the cytoplasm and plays a major role in the modification and dissolution of heat denatured protein aggregates (Parsell et al., 1994; Glover and Lindquist, 1998; Bösl et al., 2006).

It is important to note that the ClpB/Hsp104 subfamily is not able to recover and refold the majority of protein substrates without the cooperation of the Hsp70 refolding system (Glover and Lindquist, 1998). This interaction is stringently specific since disaggregation is contingent on the presence of Hsp70 and Hsp100 from the same species (Glover and Lindquist, 1998). Notably, another AAA representative found in *S. cerevisiae* is the mitochondrial 78 kDa heat shock protein, known as Hsp78. In this review, we have explored some features of Hsp78 and speculate the possible existence of other homologs in animals/metazoans.

## Hsp78

### Sequence and structure

As a representative AAA member localized to the mitochondrial matrix of *S. cerevisiae*, Hsp78 has a characteristic signal peptide at the N-terminus required for its proper subcellular localization (Leonhardt et al., 1993). This protein is 811 residues long (predicted mass of 91,336 Daltons) and shares more identity to EcClpB (about 49%) than to ScHsp104 (about 42%), likely due to its mitochondrial origin. Also, Hsp78 is shorter than both proteins because it is truncated at the N-terminus, which is involved in substrate binding in other homologs. The two NBDs are from residues 98 to 344 (NBD1) and 467 to 658 (NBD2; Figure S1). Within these domains, the two ATP binding sites are located from residues 143 to 150 and 541 to 548. The region responsible for substrate binding, located in the first AAA domain is well conserved among these chaperones, notably Tyr251 (ClpB numbering; Figure S1), which is required for binding as deemed by site-directed mutagenesis and cross-linking assays (Schlieker et al., 2004).

The NBD1 is primarily responsible for the ATPase activity, since specific mutations in these sites and others can interfere with ATPase activity (Table S1). On the other hand, the NBD2 is required for proper oligomerization. Although the preferred substrate of Hsp78 is ATP, it can also hydrolyze GTP, CTP and UTP, but with a decrease in efficiency ranging from one tenth to one fiftieth that of ATP hydrolysis (Krzewska et al., 2001a). However, it is worth mentioning that these experiments were performed at high nucleotide concentrations.

Despite no high-resolution structure for Hsp78 being available (see Leidhold et al., 2006 for a model structure), its high sequence identity with EcClpB likely implies the two proteins share a strong degree of structural resemblance. Figure 1B shows one of the available structures for EcClpB (PDB number 4CIU) from X-ray diffraction, which has a 3.5 Å resolution and covers 727 residues, from 159–247, 253–285, 294–323, 333–430, 441–649, 659–729, and 732–858 (Figure S2). To better understand why the proteins likely share structural resemblance, a model for the 4CIU structure was produced (Figure 1B). In this model, all residues that are identical between EcClpB and Hsp78, according to the alignment shown in Figure S1, are colored in black in Figure 1B. Clearly, the residues that are identical to Hsp78 occupy several positions and are almost evenly spaced throughout the protein, a strong indication that the proteins may have a similar conformation. It is also important to point out that similar residues were not included in this model, although they may also adopt a similar conformation. Since the structure for the monomer may be similar, it is just as intuitive that the quaternary structure may also be analogous. As a matter of fact, Leidhold et al. (2006) created a model structure for a hexameric Hsp78 and showed that it is very similar to the hexameric ClpB.

In this sense, is important to note that in the presence of nucleotides, ClpB changes its conformation mainly in the NBD1 domain (Figure 1C; for a review see Doyle and Wickner, 2009). Also, Hsp78 oligomerizes to form a hexamer, which influences its ATPase and chaperone activities, although in purified mitochondria smaller oligomers have been identified (Leidhold et al., 2006). Notably, the oligomerization of Hsp78 is dependent upon both protein and ATP concentrations and stoichiometries. In the presence of ATP, Hsp78 elutes with a molecular mass of a hexamer, while it elutes with a much lower apparent molecular mass in the absence of this nucleotide (Krzewska et al., 2001a). Thus, the oligomerization process depends on the concentration of Hsp78, and consequently the protein is more active at higher than lower concentrations (Krzewska et al., 2001a).

### Function

Hsp78 is expressed in the mitochondrial matrix of yeast, and its expression increases upon heat shock. Leonhardt et al. (1993) demonstrated that the number of transcripts belonging to Hsp78 increased approximately 10 fold when cells were heated for 1 h at 42°C. However, as described for Hsp104, Hsp78 is not essential for cell growth, as Hsp78 deleted yeast are viable (Leonhardt et al., 1993). Moreover, while Hsp104 is important for thermotolerance, Hsp78 appears not to play an important role in tolerance to heat (Sanchez and Lindquist, 1990; Leonhardt et al., 1993). Surprisingly, Hsp78 is capable of partially complementing induced thermotolerance of Hsp104 in an Hsp104 knock out strain, when expressed in the cytosol (Schmitt et al., 1996; Table S2).

While Hsp78 may have little or no role in conferring cellular thermotolerance, the chaperone plays important functions in the mitochondria. Deletion of Hsp78 is lethal in cells deleted (Schmitt et al., 1995) or carrying specific point-mutations in the mitochondrial Hsp70 (Moczko et al., 1995), which may suggest functional overlap between these two chaperones. In these studies, deficiency in protein import and aggregation in the matrix were detected and eliminated by the expression of Hsp78 (Moczko et al., 1995; Schmitt et al., 1995). The interaction between Hsp70 and Hsp78 has been demonstrated in several studies and Hsp78 can substitute for some chaperone functions of mitochondrial Hsp70 (Schmitt et al., 1995). The two chaperones combined are more efficient when refolding several substrates, either model or specific mitochondrial proteins (Krzewska et al., 2001b; Germaniuk et al., 2002).

Hsp78 is essential for mitochondrial thermotolerance (maintenance of respiratory competence and genome integrity under severe temperature stress) and the recovery of mitochondrial misfolded proteins after heat shock (Schmitt et al., 1996). Other experiments have demonstrated that survival under conditions in which cell growth depends on mitochondrial respiration is severely affected by the deletion of the hsp78 gene (Schmitt et al., 1996). In this case, deletion caused respiratory incompetence and lesions in mitochondrial DNA (Schmitt et al., 1996). Additionally, the presence of Hsp78 is essential in aggregation and disaggregation assays, which were performed on intact mitochondria in order to resolubilize protein heat stress induced aggregates under *in vivo* conditions (von Janowsky et al., 2006). Hsp78 is also important for the recovery of the normal morphology of yeast mitochondria after severe heat stress as deletion of Hsp78 delays the recovery (Lewandowska et al., 2006). Under stress conditions, Hsp78 cooperates with other mitochondrial heat shock proteins (Schmitt et al., 1996). This protein cooperates with the mitochondrial Hsp70 system (Hsp70/DnaJ/GrpE) to refold luciferase *in vitro* experiments (Krzewska et al., 2001b). Also, it cooperates with proteolytic systems, such the Pim1/LON complex (for proteolysis in mitochondria) (Röttgers et al., 2002). In summary, Hsp78 is a member of the Protein Quality Control (PQC) system in the matrix of yeast mitochondria that, together with the proteostatic system, is part of a network of utmost importance that protects cells against misfolding and aggregation (Douglas et al., 2009; Tiroli-Cepeda and Ramos, 2011).

### Is there a mammalian homolog?

**Figure 2:**
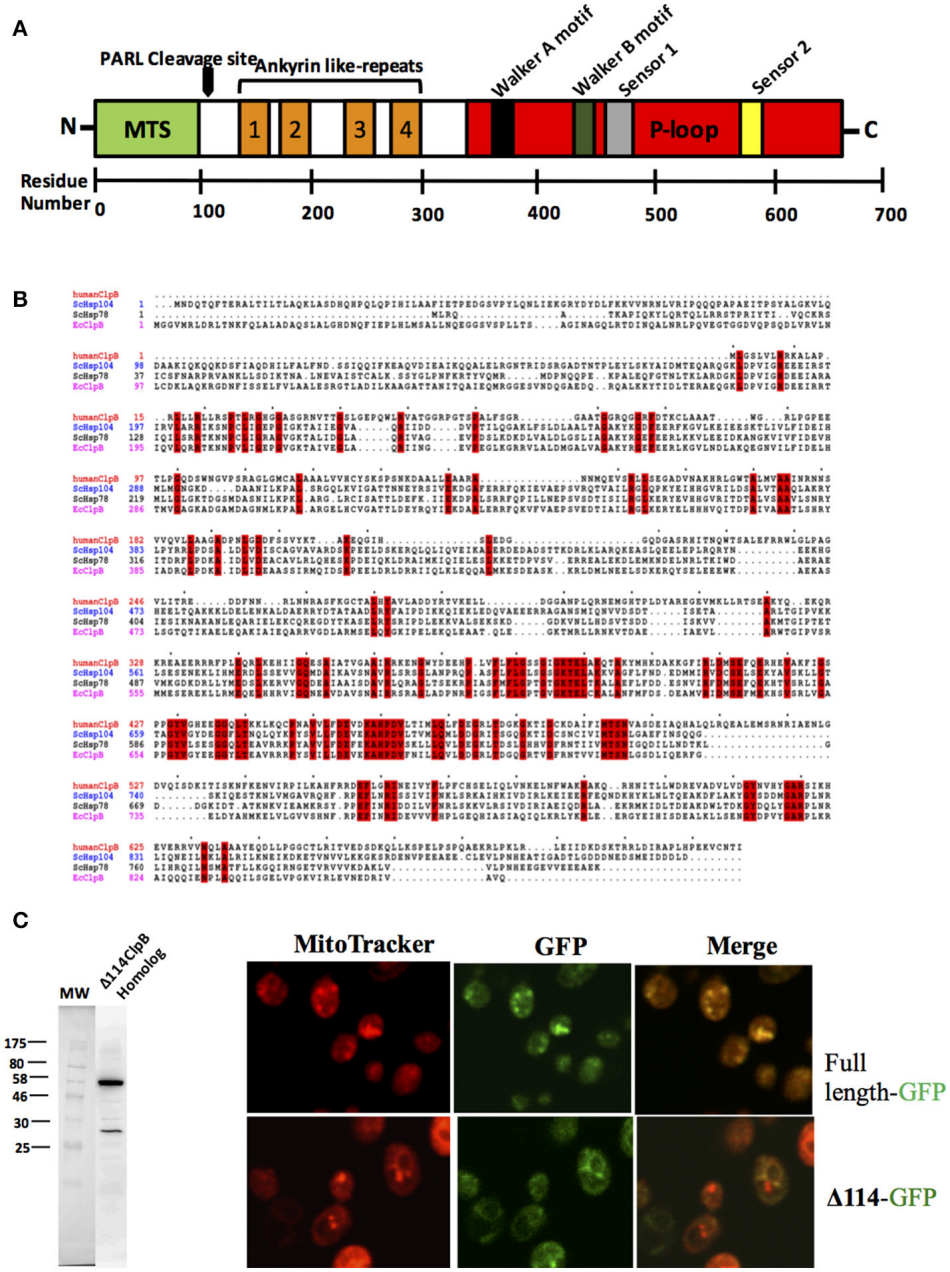
A human ClpB homolog (Q9H078)? **(A)** This gene has a signal peptide at its N-terminus for translocation (MTS), four ankyrin domains (two antiparallel α-helices followed by a β-hairpin) (in orange) and a single nucleotide binding domain (in red) containing canonical Walker A, Walker B, Sensor 1, and sensor 2 motifs. There is a rhomboid protease (PARL) cleavage site between the MTS and ankyrin like-repeats. **(B)** Amino acid sequence alignment of ClpB from *Escherichia coli*, Hsp104 from *Saccharomyces cerevisiae* and Q9H078 from human. Alignment created using Clustal (Sievers et al., 2011). **(C)** Q9H078 has GFP fluorescence precisely where mitochondria are detected, whereas the same fusion lacking the localization signal does not replicate this pattern. Left, western blot performed against human Q9H078 (dilution of 1:1000, abcam, ab87253). A homemade ECL solution was prepared for the chemiluminescent reaction, and the resulting signal was detected using a digital Chemiluminescent imaging system (GE). Right, confocal analysis of localization of the Q9H078 full length-GFP and Δ114-GFP in yeast cells. Cells were grown at 30°C and treated with the fluorescent dye MitoTracker Red (which targets active mitochondria in yeast) and subsequently fixed with 4% formaldehyde for 30 min. Cells were then washed twice with PBS (Phosphate-buffered saline) and resuspended in the same buffer and viewed by confocal microscopy (Leica, TCS SP5).

Hsp78 is not present in metazoans but there is a gene sometimes referred to as the “ClpB homolog” that has a single nucleotide binding domain containing canonical Walker A and B motifs (Figure 2A). This protein is also referred to as Q9H078 (in humans) and ANKCLP in general (Erives and Fassler, 2015). Previous results showed that Q9H078 is capable of hydrolyzing ATP when recombinantly expressed (Wortmann et al., 2015). When only the single nucleotide binding domain is considered, sequence identity with known Hsp104/ClpB members is about 40% (65% similar). However, this sequence identity decreases to about 20% when the entire sequence is considered (Figure 2B).

Similar to Hsp78, Q9H078 also has the characteristic signal peptide at the N-terminus for localization to mitochondria (Figure 2A), and its expression has been detected in cell lines derived from human and murine tissues (Périer et al., 1995; Kanabus et al., 2015; Saita et al., 2017). Heterologous expression studies in yeast demonstrate that a histidine tagged 114 N-terminal truncation of the Q9H078 will express, while expression of the complete or untagged version of the protein is not detected by western blot (Figure 2C). This could imply that, in yeast, the protein is localized to the mitochondria, where it is cleaved and subsequently degraded. In support of this, yeast transformed with a C-terminal GFP fusion of the Q9H078 have GFP fluorescence precisely where mitochondria are detected, whereas the same fusion lacking the localization signal does not depict this phenomenon (Figure 2C). Importantly, the GFP fluorescence in these experiments serves as an artifact to the presence of the Q9H078, since it was not directly detected by immunoblot analysis. It is worth noting that Hsp78 is more related to ClpB than Hsp104 due to its origin in the mitochondria. Therefore, when only considering this particular aspect, Q9H078 should be considered functionally related to Hsp78, not to Hsp104.

Of noticeable significance, certain mutations in the human Q9H078 gene are associated with a number of pathologies implicated in mitochondrial disorders, one of which can be mimicked in zebrafish but rescued when the native human gene is introduced (Kanabus et al., 2015; Wortmann et al., 2015). The protein also has four ankyrin (two antiparallel α-helices followed by a β-hairpin) domains near the N-terminus, which likely mediate protein-protein interactions (Figure 2A). Indeed, it has been shown to associate with ATP2A2 and cleaved by the rhomboid protease PARL, which are both involved in apoptosis (Wortmann et al., 2015; Saita et al., 2017). The cleavage site for PARL lies between the cysteine residue at position 126 and the tyrosine residue at position 127, which excises the localization signal while preserving the ankyrin repeats and nucleotide binding domain (Saita et al., 2017).

In any event, the loss of both hsp104 and hsp78 genes in metazoans (Erives and Fassler, 2015) is striking, and opens the debate whether or not one or more genes can have the functions of these chaperones in animals (Mokry et al., 2015; Wortmann et al., 2015). Erives and Fassler (2015) showed that the *ANKCLP* gene occurs alongside the *Hsp104* and *Hsp78* genes in choanoflagellates, indicating that it may be a fusion of an N-terminal ankyrin domain and the C-terminal domain of a *clp* gene. There are a large variety of clp groups and it is fairly difficult to point out which gene contributed to the C-terminal domain.

Despite its medical importance, ANKCLP appears not to be a bona fide replacement for the lack of Hsp78, nor Hsp104, as indicated by some factors. One is the fact that the *ANKCLP* gene lacks the NBD1 which is important for function and ATP-induced conformational changes (Figure 2B). Additionally, since the homology between NBD1 and NBD2 is limited (Schirmer et al., 1996), it would be unexpected that the presence of only one could suffice for the entire function. Another point was raised by the work of Erives and Fassler (2015), in which the region upstream of the ANKCLP gene lacks the archetypical heat shock element consensus sequences that allow multimeric binding of the transcription Heat Shock Factor 1 (Hsf1). This extragenic region is strictly conserved in organisms with genuine Hsp78 and Hsp104 genes (Erives and Fassler, 2015).

Nonetheless, the investigations discussed here imply that much work has yet to be done to determine whether or not one or more genes can have the functions of Hsp78 and Hsp104 in animals.

## Conclusion

Hsp78 is a representative member of the AAA family in the yeast mitochondria. This chaperone has ATPase activity and can oligomerize into a hexamer or smaller oligomers in isolated mitochondria in a concentration dependent manner. Also, Hsp78 is essential for proper recovery following mitochondrial stress, as the chaperone associates with other Hsps as part of the mitochondrial PQC system. The lack of an Hsp78 homolog in metazoans is enigmatic due to its important role on degradation of fungal mitochondrial proteins. Thus, whether metazoans have completely lost Hsp78/Hsp104 activities remains an open question.

## Author contributions

All authors listed have made a substantial, direct and intellectual contribution to the work, and approved it for publication.

### Conflict of interest statement

The authors declare that the research was conducted in the absence of any commercial or financial relationships that could be construed as a potential conflict of interest.
