# Supplementary material for: Hsp78 (78 kDa Heat Shock Protein), a Representative AAA Family Member Found in the Mitochondrial Matrix of Saccharomyces cerevisiae
Source: Front Mol Biosci. 2017 Aug 23;4:60. doi: 10.3389/fmolb.2017.00060 (PMC5572323; doi:10.3389/fmolb.2017.00060)

Hsp78 (78 kDa heat shock protein), a representative AAA family member found in the mitochondrial matrix of *Saccharomyces cerevisiae*. A Mini Review

Josielle Abrahão[1], David Z. Mokry[1], Carlos H. I. Ramos[1*]

[1]Chemistry Institute, University of Campinas (UNICAMP), Campinas, SP, Brazil.

*Corresponding author: email cramos@iqm.unicamp.br, phone 55-19-3521-3096.

Supplemental material

Table S1: The effect of mutations on Hsp78.

RESIDUE	MUTATION	EFFECT (compared to the wild-type)	REFERENCE
Lys149	Thr	no measurable ATPase activity; impaired reactivation of aggregated luciferase	Krzewska, et al., 2001
Lys547	Thr	8% of ATPase activity; impaired reactivation of aggregated luciferase	Krzewska, et al., 2001

Table S2: Yeast survival at 50°C in different conditions.

Strain/conditions	After 10 min	After 20 min	REFERENCE
Wild-type	~80%	~40%	Schmitt et al, 1996
ΔHsp104	~20%	~2%	Schmitt et al, 1996
ΔHsp104 and ΔHsp78	-	< 1%	Erives and Fassler, 2015
ΔHsp104; Hsp78 induction	~70%	~15%	Schmitt et al, 1996

Figure S1: Amino acid sequence alignment of ClpB from *Escherichia coli* (EcClpB) and Hsp104 (ScHsp104) and Hsp78 (ScHsp78) from *Saccharomyces cerevisiae*. Alignment created using Clustal Omega (Sievers et al., 2011) and ESPript 3.0. ScHsp104 is approximately 43% identical to EcClpB and 42% identical to ScHsp78, whereas EcClpB is about 49% identical to ScHsp78. The NBD1 is boxed in red and the NBD2 is boxed in blue.

```
ScHsp104    1  ...MNDQTQFTERALTILTLAQKLASDHQHPQLQPIHILAAFIETPEDGSVPYLQNLIEKGRYDYDLFKKVVNRNLVRIPQQQPAPAEITPSYALGKVLQ
ScHsp78     1  .........................................MLRQ...............A......TKAPIQKYLQRTQLLRRSTPRIYTI..VQCKRS
EcClpB      1  MGGVMRLDRLTNKFQLALADAQSLALGHDNQFIEPLHLMSALLNQEGGSVSPLLTS....AGINAGQLRTDINQALNRLPQVEGTGGDVQPSQDLVRVLN

ScHsp104   98  DAAKIQKQQKDSFIAQDHILFALFND.SSIQQIFKEAQVDIEAIKQQALELRGNTRIDSRGADTNTPLEYLSKYAIDMTEQARQGKLDPVIGREEEIRST
ScHsp78    37  ICSFNARPRVANKLLSDIKTNA.LNEVAISTCALK.SSYGLPNFKRTYVQMR.....MDPNQQPE..KPALEQFGTNLTKLARDGKLDPVIGRDEEIARA
EcClpB     97  LCDKLAQKRGDNFISSELFVLAALESRGTLADILKAAGATTANITQAIEQMRGGESVNDQGAEDQ..RQALKKYTIDLTERAEQGKLDPVIGRDEEIRRT

ScHsp104  197  IRVLARRIKSNPCLIGEPGIGKTAIIEGVAQRIIDDDVPTILQGAKLFSLDLAALTAGAKYKGDFEERFKGVLKEIEESKTLIVLFIDEIHMLMGNGKD.
ScHsp78   128  IQILSRRTKNNPCLIGRAGVGKTALIDGLAQRIVAGEVPDSLKDKDLVALDLGSLIAGAKYRGEFEERLKKVLEEIDKANGKVIVFIDEVHMLLGLGKTD
EcClpB    195  IQVLQRRTKNNPVLIGEPGVGKTAIVEGLAQRIINGEVPEGLKGRRVLALDMGALVAGAKYRGEFEERLKGVLNDLAKQEGNVILFIDELHTMVGAGKAD

ScHsp104  296  ...DAANILKPALSRGQLKVIGATTNNEYRSIVEKDGAFERRFQKIEVAEPSVRQTVAILRGLQPKYEIHHGVRILDSALVTAAQLAKRYLPYRRLPDSA
ScHsp78   228  GSMDASNILKPKLARG.LRCISATTLDEFK.IIEKDPALSRRFQPILLNEPSVSDTISILRGLKERYEVHHGVRITDTALVSAAVLSNRYITDRFLPDKA
EcClpB    295  GAMDAGNMLKPALARGELHCVGATTLDEYRQYIEKDAALERRFQKVFVAEPSVEDTIAILRGLKERYELHHHVQITDPAIVAAATLSHRYIADRQLPDKA

ScHsp104  393  LDLVDISCAGVAVARDSKPEELDSKERQLQLIQVEIKALERDEDADSTTKDRLKLARQKEASLQEELEPLRQRYNEEKHGHEELTQAKKKLDELENKALD
ScHsp78   326  IDLVDEACAVLRLQHESKPDEIQKLDRAIMKIQIELESLKKETDPVSV..ERREALEKDLEMKNDELNRLTKIWDAERAEIESIKNAKANLEQARIELEK
EcClpB    395  IDLIDEAASSIRMQIDSKPEELDRLDRRIIQLKLEQQALMKESDEASK..KRLDMLNEELSDKERQYSELEEEWKAEKASLSGTQTIKAELEQAKIAIEQ

ScHsp104  493  AERRYDTATAADLRYFAIPDIKKQIEKLEDQVAEEERRAGANSMIQNVVDSDTISETAARLTGIPVKKLSESENEKLIHMERDLSSEVVGQMDAIKAVSN
ScHsp78   424  CQREGDYTKASELRYSRIPDLEKKVALSEKSKD.....GDKVNLLHDSVTSDDISKVVAKMTGIPTETVMKGDKDRLLYMEDSLKERVVGQDEAIAAISD
EcClpB    493  ARRVGDLARMSELQYGKIPELEKQLEAAT.QLE.....GKTMRLLRNKVTDAEIAEVLARWTGIPVSRMMESEREKLLRMEQELHHRVIGQNEAVDAVSN

ScHsp104  593  AVRLSRSGLANPRQP.ASFLFLGLSGSGKTELAKKVAGFLFNDEDMMIRVDCSELSEKYAVSKLLGTTAGYVGYDEGGFLTNQLQYKPYSVLLFDEVEKA
ScHsp78   519  AVRLQRAGLTSEKRPIASFMFLGPTGTGKTELTKALAEFLFDDESNVIRFDMSEFQEKHTVSRLIGAPPGYVLSESGGQLTEAVRRKPYAVVLFDEFEKA
EcClpB    587  AIRRSRAGLADPNRPIGSFLFLGPTGVGKTELCKALANFMFDSDEAMVRIDMSEFMEKHSVSRLVGAPPGYVGYEEGGYLTEAVRRRPYSVILLDEVEKA

ScHsp104  692  HPDVLTVMLQMLDDGRITSGQGKTIDCSNCIVIMTSNLGAEFINSQQG.....SKIQESTKNLVMGAVRQHFRPEFLNRISSIVIFNKLSRKAIHKIVDI
ScHsp78   619  HPDVSKLLLQVLDEGKLTDSLGHHVDFRNTIIVMTSNIGQDILLNDTKLGDDGKIDT.ATKNKVIEAMKRSYPPEFINRIDDILVFNRLSKKVLRSIVDI
EcClpB    687  HPDVFNILLQVLDDGRLTDGQGRTVDFRNTVVIMTSNLGSDLIQERFG.....ELDYAHMKELVLGVVSHNFRPEFINRIDEVVVFHPLGEQHIASIAQI

ScHsp104  787  RLKEIEERFEQNDKHYKLNLTQEAKDFLAKYGYSDDMGARPLNRLIQNEILNKLALRILKNEIKDKETVNVVLKKGKSRDENVPEEAEECLEVLPNHEAT
ScHsp78   718  RIAEIQDRLA..EKRMKIDLTDEAKDWLTDKGYDQLYGARPLNRLIHRQILNSMATFLLKGQIRNGETVRVVVKDAKLV.............VLPNHEEG
EcClpB    782  QLKRLYKRLE..ERGYEIHISDEALKLLSENGYDPVYGARPLKRAIQQQIENPLAQQILSGELVPGKVIRLEVNEDRIV.............AVQ.....

ScHsp104  887  IGADTLGDDDNEDSMEIDDDLD
ScHsp78   803  EVVEEEAEK.............
EcClpB         ......................
```

Figure S2: Structure from EcClpB (PDB number 4CIU) (Carroni et al., 2014) covers 727 residues, from 159-247, 253-285, 294-323, 333-430, 441-649, 659-729 and 732-858. Figure from http://www.rcsb.org/pdb/explore/remediatedSequence.do?structureId=4CIU.

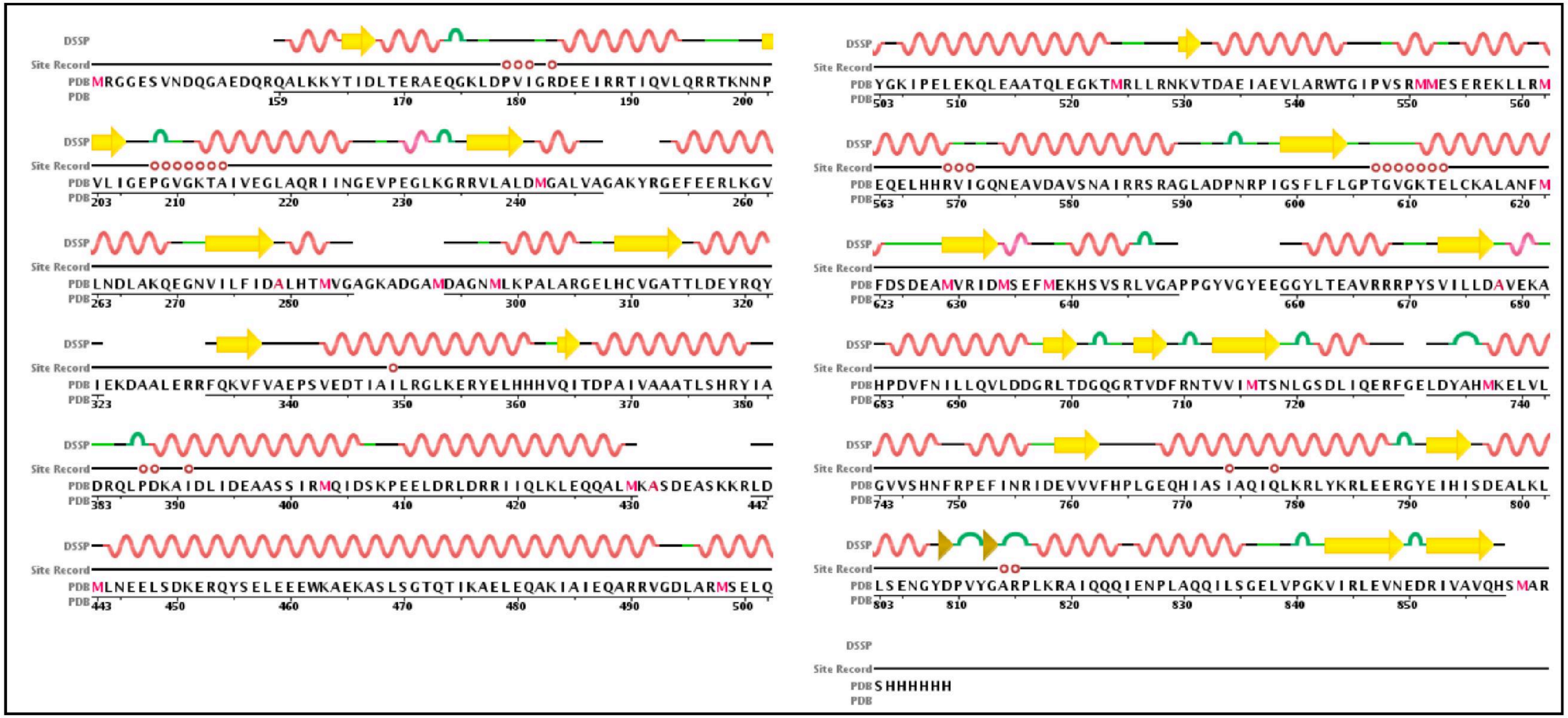

Supplement: Supplementary file 1 [file Presentation1.PDF]
